# Exploring Socio-Behavioral Correlates of Metabolic and Inflammatory Risk in a University Sample Residing Along the U.S./Mexico Border: A Pilot Study Concomitantly Collecting Survey Data, Blood and Hair Samples, and Physical Measures

**DOI:** 10.3390/ijerph22040647

**Published:** 2025-04-20

**Authors:** Gabriel A. Frietze, Cai Xu, Bibiana Mancera, Elisa Robles-Escajeda, Alyssa A. Martinez, Michelle Gil, Diana P. Flores, Khodeza Begum, Panfeng Liang, Abhijit Mandal, Michael Nsiah-Nimo, Nilotpal Sanyal, Ming-Ying Leung, Michael J. Kenney, Robert A. Kirken

**Affiliations:** Border Biomedical Research Center, College of Science, University of Texas at El Paso, 500 West University Avenue, El Paso, TX 79968, USA; cxu@utep.edu (C.X.); barias@utep.edu (B.M.); erobles3@utep.edu (E.R.-E.); aamartinez18@utep.edu (A.A.M.); mgil@miners.utep.edu (M.G.); dpflores@utep.edu (D.P.F.); kbegum@utep.edu (K.B.); pliang2@utep.edu (P.L.); amandal@utep.edu (A.M.); mnsiahnimo@utep.edu (M.N.-N.); nsanyal@utep.edu (N.S.); mleung@utep.edu (M.-Y.L.); mjkenney@utep.edu (M.J.K.); rkirken@utep.edu (R.A.K.)

**Keywords:** Hispanic, inflammation, diabetes, body mass index, HbA1c

## Abstract

Hispanic adults have an increased incidence of type 2 diabetes (T2D) at a younger age and diagnosis of certain cancers, including liver, stomach, and colorectal, which may be attributed to metabolic health. Several key metabolic health indicators, such as hemoglobin A1c (HbA1c), body mass index (BMI), and waist-to-hip ratio (WHR), have been linked to obesity. The purpose of this pilot study was to explore the complex relationships between socio-behavioral factors that lead to the increased incidence of metabolic syndrome (e.g., HbA1c) and chronic inflammation (interleukins) in Hispanics. Two hundred and twelve Hispanic participants (*M*_age_ = 43.45, *SD* = 15.36) who identified predominantly as female (72.17%) were included in the study. Correlational analyses revealed that HbA1c was positively associated with age and negatively associated with several socio-behavioral factors, including overall health, quality of life, physical health, physical performance, social support, mother’s education, and father’s education. These findings highlight the importance of social support and parental involvement in diabetes management. The focused integration of socio-behavioral and biological data provides a powerful foundation for future research and the development of targeted interventions.

## 1. Introduction

Metabolic health disparities in Hispanics warrant further investigation, as Hispanic adults have an increased incidence of type 2 diabetes (T2D) at a younger age [1] and diagnosis of certain cancers, including liver, stomach, and colorectal [2,3,4], which may be attributed to metabolic health. Several key metabolic health indicators, such as hemoglobin A1c (HbA1c), body mass index (BMI), and waist-to-hip ratio (WHR), have been linked to obesity. Recent studies suggest that elevated HbA1c levels [5,6,7], insulin resistance [8,9,10], and WHR [11,12] are associated with an increased risk for metabolic syndrome [13,14] and the development of chronic diseases [15,16,17,18,19], which result in poor health outcomes. Interleukins (IL-1 and IL-6) and cortisol are key in immune-inflammatory responses to stress in the body. Interleukins (ILs) are important to study, given their dual role in inflammation regulation. ILs are the signaling molecules that regulate immune system responses by assisting in the activation and recruitment of immune cells, while anti-inflammatory ILs can aid in suppressing immoderate inflammation and promote repairing tissue [20]. Importantly, chronic stress can lead to prolonged cortisol production that suppresses the immune system and over extended periods of time causes inflammation, increasing the risk for inflammatory diseases such as obesity [21], diabetes [22], cancer [23,24,25], cardiovascular disease [26,27,28], and autoimmune disorders [29]. Hair cortisol analysis affords an opportunity to assess cortisol production over a longer period instead of at a single point in time using saliva or blood serum [30].

The association between socio-behavioral factors and metabolic health has been established. Furman et al. (2019) revealed that systemic chronic inflammation leads to the incidence of cancer, cardiovascular disease (CVD), and diabetes mellitus (DM), and is mediated by socio-behavioral factors [31]. Additionally, van Zyl et al. (2020) reported increased inflammatory biomarkers such as fibrinogen (a protein) and C-reactive protein among people with chronic diseases such as CVD and DM, and were heavily influenced by modifiable behaviors such as diet, exercise, alcohol consumption, and smoking [32]. However, a dearth of knowledge exists regarding the correlation between socio-behavioral factors and metabolic health among Hispanics who also experience health disparities.

Health disparities are the differences between health outcomes in two populations and include the prevalence of disease, health status, life expectancy, and mortality [33] and are influenced by social determinants of health (SDH), defined as where a person is born, lives, is educated, works, worships, recreates, and ages. In marginalized and under-resourced communities in the United States (U.S.), these SDH exert a significant influence on obesity, cardiovascular disease, and T2D [34,35] and exacerbate health disparities. There is a critical need for studies to examine the multiple factors that affect metabolic and inflammatory health to yield a more comprehensive understanding of the socio-behavioral correlates of metabolic health in Hispanics that can inform the development of targeted interventions to reduce Hispanic health disparities. Studies examining multiple factors such as diet, physical activity, obesity, sleep, social support, stress, smoking, and the physical/built environment that affect metabolic and inflammatory health are critically needed among Hispanics to yield a more comprehensive understanding of the socio-behavioral correlates of metabolic health that can inform the development of targeted interventions to reduce Hispanic health disparities.

### Theoretical Framework

The National Institute on Minority Health and Health Disparities (NIMHD) framework was utilized to conceptually frame this research study within the context of the SDH and health disparities that affect Hispanics of Mexican origin [36]. This facilitated an in-depth analysis of factors that impact under-resourced populations. The NIMHD framework illustrates the complex interactions between the five domains of influence (biological, behavioral, physical/built environment, socio-cultural environment, and health care system) and the four levels of influence (individual, interpersonal, community, and societal). The current study focused on the individual level of influence across four domains of influence: biological (e.g., cytokines), behavioral (e.g., education), physical/built environment (neighborhood), and sociocultural environment. The strategic integration of socio-behavioral factors, inflammation biomarkers, and physical assessment provides a novel and innovative strategy for understanding metabolic and inflammatory indicators in Hispanics.

Hispanic health disparities persist through the complex interactions between socio-behavioral, environmental, and physiological factors. The purpose of this small-scale pilot study was to explore the complex relationships between socio-behavioral factors that lead to the increased incidence of metabolic syndrome (e.g., HbA1c) and chronic inflammation (interleukins) in Hispanics. We hypothesized that metabolic indicators (HbA1c, BMI, WHR) will be negatively associated with overall health, quality of life, physical health, physical performance, and social support, and positively associated with age and residing in a disordered neighborhood. Findings from the current pilot study will be used to refine our research methods and assess the feasibility of implementing this study in larger samples and longitudinally.

## 2. Materials and Methods

### 2.1. Participants

Two hundred and twelve Hispanic participants (*M*_age_ = 43.45, *SD* = 15.36) who identified predominantly as female (72.17%) were included in the study. Informed consent was obtained from all study participants. Students, faculty, and staff were recruited from the University of Texas at El Paso (UTEP) to participate. Eligibility requirements included being: (1) 18 years of age or older, (2) a UTEP student, faculty, or staff, (3) Hispanic, (4) willingness to provide a blood sample (as outlined in the consent form: approximately 10–15 mL of blood), and (5) willingness to provide a hair sample of approximately 30–50 strands of at least 3 cm in length. Research protocols, recruitment, and participation of human subjects were approved by UTEP’s Institutional Review Board (IRB) in accordance with relevant guidelines and regulations.

### 2.2. Measures

#### 2.2.1. Demographics and Background Questionnaire

A nine-item demographics and backgrounds survey examined age, sex, race, marital status, neighborhood, income, education, mother’s education, and father’s education.

#### 2.2.2. Socio-Behavioral Instruments

Participants completed inventories from the National Institutes of Health (NIH) *All of Us* Research Program, which included items under the categories of “Overall Health” (assessing general health status), “Neighborhood” (assessing neighborhood environment), and “Supportive Relationships” (assessing social interactions) [37]. These measures utilize Likert-type scales to capture participant responses. See Table 1 for sample item.

#### 2.2.3. The Multidimensional Scale of Perceived Social Support (MSPSS)

The MSPSS is a self-report scale consisting of twelve items used to assess various dimensions of social support that include family, friends, and a significant other [38]. Participants rated each item using a 7-point Likert scale ranging from “very strongly disagree” to “very strongly agree”. In the current study, the mean total score of the MSPSS was examined, with a higher score indicating a higher level of perceived social support. See Table 1 for sample item.

#### 2.2.4. The Modified Medical Outcomes Study Social Support Survey (mMOS-SS)

The mMOS-SS comprises two dimensions: instrumental and emotional social support [39]. In this study, the instrumental social support score was used and calculated as the mean of four items, ranging from 1 (none of the time) to 5 (all of the time). A higher score (closer to 5) indicates that the individual consistently perceives strong support from others in tasks such as transportation to the doctor, meal preparation, and assistance with daily chores when needed.

#### 2.2.5. The (Ross–Mirowsky) Neighborhood Physical Disorder Scale

The (Ross–Mirowsky) Neighborhood Physical Disorder Scale is a self-report scale consisting of twelve items to assess individuals’ perceptions of disorder within their neighborhoods [40]. These items address visible signs of physical decay (e.g., graffiti, abandoned buildings) and social disorder (e.g., crime, substance and alcohol abuse). Participants rated each item using a 4-point Likert scale ranging from “strongly disagree” to “strongly agree”. The mean score of the six items on this scale was used in this study, with a score range of 1–4. See Table 1 for sample item.

#### 2.2.6. Metabolic and Inflammatory Health Indicators

Participants were asked to provide physical measures (i.e., weight, height, and waist and hip circumference), as well as blood and hair samples. BMI and WHR were measured by standard techniques, with higher levels of BMI and WHR indicating poor metabolic health. Blood was extracted from overnight fasting participants using EDTA tubes. Whole blood samples were utilized to assess HbA1c with the Abbot Afinion HbA1c point-of-care analyzer. EDTA blood sample tubes were centrifuged at 1000× *g* within 30 min of collection. Plasma was removed, aliquoted, and stored at −80 °C. Luminex multiplex assays employing xMAP (Multi-Analyte Profiling) bead-based technology in EDTA plasma were utilized to detect and quantify a panel of inflammatory cytokines (interleukins: IL-1a, IL-1b, IL-1ra, IL-2, IL-4, IL-5, IL-6, IL-7, IL-8, IL-10, IL-12p40, IL-12p70, IL-13, IL-15, IL-17f) using MILLIPLEX Human Cytokine/Chemokine/Growth Factor Panel A kit (intra-assay 15% CV and inter-assay within 20% CV). Samples were run in duplicate along with standards and quality controls. Data analysis was performed using the Belysa Curve Fitting Software version 1.2. Elevated HbA1c and interleukin levels serve as indicators of poor metabolic health. Hair samples were used to measure chronic cortisol levels via LC-MS as previously described [41]. Hair was always collected from the posterior vertex to reduce variability in cortisol distribution, and consistent hair length (~3 cm) was used to represent cortisol levels over a fixed time frame. Inter- and intra-assay variability was systematically assessed to maintain reproducibility. Internal standards, quality controls, and calibration curves were included in every run.

### 2.3. Procedure

Participants began by reading and signing an electronic consent form, which set expectations that the participant needed to schedule an in-person appointment at a prespecified location on campus after completing the survey in order to obtain physical assessments, blood, and hair samples. Participants were then assigned a unique identifier to pair survey instruments and the biospecimens to create one participant file. Participants then completed a 30–45 min survey assessing socio-behavioral factors, which could be completed online or in-person at the prespecified location. If participants opted to complete the survey in person, they were taken to a quiet room to complete the survey via a laptop or tablet provided by the investigators. After completing the survey, participants promptly scheduled an appointment to complete the remainder of the study at the Center for Integrated and Translational Research, which includes a clinical laboratory. Upon arrival at their scheduled appointment, participants were asked to complete a physical assessment (height, weight, waist, and hip circumference) and provide biospecimens (blood and hair samples) which were collected by certified staff and labeled with the participant’s unique identifier. After participants provided blood samples, they were escorted to a waiting room where they were seated and provided with snacks and drinks, and asked to remain seated for 15 min while a team member observed them for any adverse effects. As participants waited, a health fact sheet explaining their results was generated and provided to them. The health fact sheet included information about the interpretation of WHR and BMI as health indicators, as well as the importance of HbA1c in assessing blood sugar control. Participants also received their HbA1c results. Lastly, participants were compensated USD 100 for their time and participation in the study. De-identified biospecimens were collected and processed by research study staff according to UTEP IRB approved protocols.

### 2.4. Statistical Analysis

Descriptive statistics were calculated for demographic and background variables. Table 2 below provides total responses and percentages for these variables. Pearson correlation coefficients were calculated to examine relationships between metabolic health indicators (HbA1c, BMI, WHR) and the continuous socio-behavioral factors. Spearman correlation coefficients were calculated to examine relationships between metabolic health indicators and the ordinal socio-behavioral factors. Lastly, Pearson correlation analyses examined associations between metabolic health indicators and a panel of inflammatory cytokines (interleukins: IL.1a, IL.1b, IL.1ra, IL.2, IL.4, IL.5, IL.6, IL.7, IL.8, IL.10, IL.12p40, IL.12p70, IL.13, IL.15, IL.17f). The percentage of missing values for all included variables ranged up to 26.89% (IL-2). Pairwise deletion was automatically applied in the correlation analysis, meaning that each pairwise correlation was calculated based on observations where both variables had available data.

## 3. Results

A majority (89%) (*n* = 190) of the sample identified as White-Latino, 4.72% identified as Indigenous Latino/a, 1.42% identified as Asian-Latino/a, 0.47% identified as Black-Latino/a, and 3.77% identified as “Other”. Forty percent (*n* = 86) of our sample reported to be married, 80% (*n* = 171) reported to live in a single-family home, 43.4% (*n* = 92) reported to have an annual income under USD 50,000.00, and more than half of the sample (63.2%, *n* = 134) were college graduates. The majority of the sample reported that their parents’ education level was below a college degree. See Table 2 for demographic and background characteristics of the participants.

### 3.1. Associations Between Metabolic Health Indicators and Socio-Behavioral Factors

Correlational analyses revealed small negative correlations between HbA1c and the following socio-behavioral factors: overall health (*r* = −0.22, *p* < 0.001), quality of life (*r* = −0.14, *p* < 0.05), physical health (*r* = −0.16, *p* < 0.05), physical performance (*r* = −0.24, *p* < 0.001), social support as indexed by the MSPSS (*r* = −0.15, *p* < 0.05), mother’s education (*r* = −0.32, *p* < 0.001), and father’s education (*r* = −0.34, *p* < 0.001). HbA1c was positively associated with age (*r* = 0.31, *p* < 0.001). See Table 3 and Table 4 for correlation analyses or Supplementary Materials for Figures S1 and S2, which include correlation heat maps.

Correlational analyses revealed that BMI was negatively associated with the following socio-behavioral factors: overall health (*r* = −0.42, *p* < 0.001), quality of life (*r* = −0.36, *p* < 0.001), physical health (*r* = −0.43, *p* < 0.001), physical performance (*r* = −0.23, *p* < 0.001), instrumental social support (*r* = −0.15, *p* < 0.05), mother’s education (*r* = −0.23, *p* < 0.001), and father’s education (*r* = −0.30, *p* < 0.001). BMI was positively associated with age (*r* = 0.36, *p* < 0.001) and neighborhood disorder (*r* = 0.16, *p* < 0.05). See Table 3 and Table 4 for correlation analyses or Supplementary Materials for Figures S1 and S2, which include correlation heat maps.

Correlational analyses revealed that WHR was negatively associated with the following socio-behavioral factors: instrumental social support (*r* = −0.19, *p* < 0.001), social support as indexed by the MSPSS (*r* = −0.22, *p* < 0.01), mother’s education (*r* = −0.27, *p* < 0.001), and father’s education (*r* = −0.28, *p* < 0.001). WHR was positively associated with age (*r* = 0.31, *p* < 0.001) and neighborhood disorder (*r* = 0.20, *p* < 0.01). See Table 3 and Table 4 for correlation analyses or Supplementary Materials for Figures S1 and S2, which include correlation heat maps.

### 3.2. Socio-Behavioral Associations

Correlations among socio-behavioral variables ranged from −0.30 to 0.80. The strongest positive correlation was between self-reported overall health and self-reported physical health (*r* = 0.80, *p* < 0.001). The strongest negative correlation was between social support as indexed by the MSPSS and neighborhood physical disorder (*r* = −0.30, *p* < 0.001). See Table 3 and Table 4 for correlation analyses or Supplementary Materials for Figures S1 and S2, which include correlation heat maps.

### 3.3. Associations Between Metabolic and Inflammatory Health Indicators

Correlation analyses examined associations between metabolic health indicators (HbA1c, BMI, and WHR) and inflammatory health indicators (interleukins). Only five statistically significant correlations emerged: (1) HbA1c was negatively correlated with IL.1a (*r* = −0.20, *p* = 0.007), (2) BMI was negatively correlated with IL.1a (*r* = −0.20, *p* = 0.007) and IL17f (*r* = −0.15, *p* = 0.045), and (3) WHR was negatively correlated with IL1a (*r* = −0.17, *p* = 0.023) and IL.5 (*r* = −0.16, *p* = 0.02). Given that these were the only five statistically significant associations that emerged between metabolic and inflammatory health indicators, we provide the associations between the 15 interleukins in Table 5 as a correlation matrix and in Supplementary Figure S3 as a correlation heatmap. Correlations among interleukins ranged from 0.17 to 0.87. The strongest correlation was between interleukins IL.1b and IL.2 (*r* = 0.87, *p* < 0.001).

## 4. Discussion

The current study’s demographic findings are seemingly representative of El Paso’s population, except for education, given that recruitment occurred in a university setting. Over half of the participants (63.2%) were college graduates, compared to 27.5% of El Paso’s population with a bachelor’s degree or higher [42]. The majority of participants in our study were of Latino origin (89%), which is slightly higher than the 82.8% of El Paso’s population that identifies as Hispanic [42]. Additionally, nearly half of the participants reported an annual income below USD 50,000, whereas the median household income in El Paso is USD 58,734 [42].

Several correlations were found in the current study, highlighting the relationship between socio-behavioral and metabolic factors. Notably, higher HbA1c levels were negatively associated with several of the socio-behavioral factors, including overall health, quality of life, physical health and performance, and social support. These findings are supported by a systematic review conducted by Pérez-Fernández et al. (2023), which identified 15 studies that explored well-being and HbA1c levels in adults with type 1 diabetes (T1D) [43]. Importantly, 11 of the studies indicated a relationship between higher HbA1c levels and lower cognitive well-being. Similarly, in a longitudinal study conducted among adults with T2D, researchers found that men with diabetes who reported higher social isolation also had higher HbA1c and blood sugar levels [44].

Another important finding that emerged in the current study is related to parental education. Lower parental education levels were associated with higher HbA1c levels, BMI, and WHR. Strikingly, this finding is partially supported by a study suggesting that higher parental education, particularly mothers’ education, was associated with lower HbA1c levels and increased parental support in adolescents with T1D [45]. Another study conducted by Zhang et al. (2024) found that blood glucose levels were negatively associated with parental economic status [46]. These findings highlight the importance of social support and parental involvement in diabetes management.

Additional findings from the study examining interleukins reveal a negative correlation between HbA1c and IL-1α, although there is a dearth of research specifically supporting this relationship. Notably, despite a statistically significant correlation emerging between HbA1c and IL-1α, the small effect size observed suggests that there are limited clinical implications for disease management or patient care. Moreover, BMI was negatively correlated with IL-1α; however, research on this relationship shows varying results. For example, in a study by Um et al. (2011), which assessed whether polymorphisms in IL-1α contribute to BMI in humans, the researchers identified an association between IL-1α gene polymorphisms and BMI in obese women [47]. In contrast, a study by Jung et al. (2010) investigating the levels of IL-1 family cytokines and the influence of obesity found no significant correlation between BMI and IL-1α levels [48]. Furthermore, WHR was also negatively correlated with IL-1α and IL-5. Research on this relationship indicates mixed results, with some studies reporting no significant association or even a positive correlation, particularly with IL-5. Research on the relationship between WHR and IL-1α is limited. For instance, a study by El-Wakkad et al. (2013), examining proinflammatory and anti-inflammatory cytokines in obese individuals, reported a positive correlation between WHR and IL-5 levels [49]. Finally, the current study found the strongest correlation between IL-1β and IL-2, suggesting that these two inflammatory markers are highly related. A study by Liu (2010) examining dynamic changes in circulatory cytokine and chemokine levels during the early phase of sepsis also found a significant positive correlation between IL-1β and IL-2 [50].

Lastly, a positive correlation between HbA1c levels and age emerged in the current study. Similarly, in a study by Gülsen et al. (2023), participants were divided into age groups, and HbA1c levels were positively associated with age for both males and females among all groups [51]. These findings were expected given that age is associated with decreases in red blood cell turnover rates and studies have found that age increases HbA1c levels independently of glucose levels and insulin resistance [52]. Future studies should further explore the associations between age and HbA1c through mediation and moderation analyses to understand the socio-behavioral factors that contribute to the positive associations observed.

Findings from this research study provide a framework from which to assess socio-behavioral survey data, biomarkers derived from blood and hair samples, and physical measures (HbA1c, BMI, WHR) to explore correlates of metabolic and inflammatory risks in Hispanics of Mexican origin living along the U.S./Mexico border. Importantly, study findings can inform clinicians about the importance of compiling a more extensive patient health record that can lead to personalized interventions to improve health outcomes. Lastly, study findings have implications for stakeholders and policymakers by providing evidence of the complex interactions between the built environment and physiological functions that drive the prevalence and incidence of diseases that affect underserved populations.

There were several limitations pertaining to this study. The first limitation is that a cross-sectional design was used in the current study, thus, causal relationships cannot be inferred from our findings. An additional limitation is related to the homogeneous sample of Hispanics of Mexican origin. This was intentional as 84% of the university’s student and staff demographic are of Mexican origin and reflect the broader community-at-large demographic of 82.8%. Another limitation was the use of a convenience sample, and all study participants were recruited from the university. This was intentional because this was a feasibility study to assess our team’s ability to conduct clinical research within a university setting and not a medical school, clinic, or hospital. Another limitation involves having a small sample size, which limits associations that can be detected and the types of analyses that can be conducted. As the sample size increases, subsequent research will incorporate comprehensive analytical approaches such as principal component analysis, latent class analysis, latent profile analysis, cluster analysis, multiple regression, or machine learning to enhance the analysis. Additionally, our socio-behavioral factors were assessed using tools that rely on subjective ratings and can introduce self-report bias. Future studies should consider objective measures, including social media analysis, to strengthen the validity of findings. Lastly, the current study did not account for a participant’s daily diet, medication history, and physical activity. These assessments should be included in future studies as they may have an impact on HbA1c.

## 5. Conclusions

The focused integration of socio-behavioral and biological data provides a powerful foundation for future research and the development of targeted interventions. The identification of specific biomarkers associated with socio-behavioral patterns can inform preventative measures and tailored treatments, particularly for populations at risk of chronic conditions influenced by both behavioral and biological factors. Collectively, the proposed pilot study substantially extends the current state of assessing socio-behavioral factors and inflammation biomarkers concomitantly, and it highlights the importance of considering both individual and systemic factors in health outcomes. Future research should explore the effects of socio-behavioral influences on inflammation biomarkers over time, as well as the potential for cross-population comparisons to better understand the role of cultural, environmental, and socio-economic factors. Future research should also expand sample diversity by including participants with a wider range of demographics (e.g., education levels) to increase the generalizability of findings. Deepening mechanism research is also needed in future studies by combining genomics or environmental exposure omics to help provide a comprehensive understanding of the influence of pollutants and other environmental stressors that might impact these biological pathways.

## Figures and Tables

**Table 1:** Descriptions of variables.

Demographic Variables	Sample Item and Response Options
Age	Sample item: What is your age in years?
Sex	Sample item: What was your biological sex assigned at birth?
	0 = Female
	1 = Male
Race	Sample item: What races do you identify with? (Select all that apply)
	1 = White-Latino/a
	2 = Black-Latino/a
	3 = Asian-Latino/a
	4 = Indigenous Latino/a
	5 = Other
Marital Status	Sample item: What is your current marital status?
	1 = Married
	2 = Divorced
	3 = Widowed
	4 = Separated
	5 = Never married
	6 = Living with partner
PANES	Sample item: What is the main type of housing in your neighborhood?
	1 = Detached single-family housing
	2 = Townhouses, row houses, apartments, or condos of 2–3 stories
	3 = Mix of single-family residences and townhouses, row houses, apartments or condos
	4 = Apartments or condos of 4–12 stories
	5 = Apartments or condos of more than 12 stories
Income	Sample item: What is your annual household income from all sources?
	1 = Less than USD 10,000
	2 = USD 10,000–USD 24,999
	3 = USD 25,000–USD 34,999
	4 = USD 35,000–USD 49,999
	5 = USD 50,000–USD 74,999
	6 = USD 75,000–USD 99,999
	7 = USD 100,000–USD 149,999
	8 = USD 150,000–USD 199,999
	9 = USD 200,000 or more
Education	Sample item: What is the highest grade or year of school you completed?
	1 = Never attended school or only attended kindergarten
	2 = Grades 1 through 4 (primary)
	3 = Grades 5 through 8 (middle school)
	4 = Grades 9 through 11 (some high school)
	5 = Grade 12 or GED (high school graduate)
	6 = 1 to 3 years after high school (some college, associate’s degree, or technical school)
	7 = College 4 years or more (college graduate)
	8 = Advanced degree (master’s, doctorate, etc.)
Mother’s Education	Sample item: What is the highest grade or year of school your mother completed?
	1 = Never attended school or only attended kindergarten
	2 = Grades 1 through 4 (primary)
	3 = Grades 5 through 8 (middle school)
	4 = Grades 9 through 11 (some high school)
	5 = Grade 12 or GED (high school graduate)
	6 = 1 to 3 years after high school (some college, associate’s degree, or technical school)
	7 = College 4 years or more (college graduate)
	8 = Advanced degree (master’s, doctorate, etc.)
Father’s Education	Sample item: What is the highest grade or year of school your father completed?
	1 = Never attended school or only attended kindergarten
	2 = Grades 1 through 4 (primary)
	3 = Grades 5 through 8 (middle school)
	4 = Grades 9 through 11 (some high school)
	5 = Grade 12 or GED (high school graduate)
	6 = 1 to 3 years after high school (some college, associate’s degree, or technical school)
	7 = College 4 years or more (college graduate)
	8 = Advanced degree (master’s, doctorate, etc.)
Overall Health	Sample item: In general, would you say your health is:
	1 = Poor
	2 = Fair
	3 = Good
	4 = Very Good
	5 = Excellent
Quality of Life	Sample item: In general, would you say your quality of life is:
	1 = Poor
	2 = Fair
	3 = Good
	4 = Very Good
	5 = Excellent
Physical Health	Sample item: In general, how would you rate your physical health?
	1 = Poor
	2 = Fair
	3 = Good
	4 = Very Good
	5 = Excellent
Physical Performance	Sample item: To what extent are you able to carry out your everyday physical activities such as walking, climbing stairs, carrying groceries, or moving a chair?
	1 = Not at all
	2 = A little
	3 = Moderately
	4 = Mostly
	5 = Completely
Instrumental Social Support Score	Sample item: Someone to help with daily chores if you were sick.
	Scoring rule for each question:
	1 = None of the time
	2 = A little of the time
	3 = Some of the time
	4 = Most of the time
	5 = All of the time
The Multidimensional Scale of Perceived Social Support (MSPSS)	Sample item: There is a special person who is around when I am in need.
	Scoring rule for each question:
	1 = Very Strongly Disagree
	2 = Strongly Disagree
	3 = Mildly Disagree
	4 = Neutral
	5 = Mildly Agree
	6 = Strongly Agree
	7 = Very Strongly Agree
Neighborhood Physical Disorder Score	Sample item: Vandalism is common in my neighborhood.
	4 = “Strongly agree
	3 = “Agree”,
	2 = “Disagree
	1 = “Strongly disagree”
Cortisol	Chronic Hair Cortisol
IL.1a, IL.1b, IL.1ra, IL.2, IL.4, IL.5, IL.6, IL.7, IL.8, IL.10, IL.12p40, IL.12p70, IL.13, IL.15, IL.17f	Cytokine biomarkers obtained from blood samples

**Table 2:** Demographic and background characteristics (*N* = 212).

Variable		Total Responses	Percentage of Responses
Sex	0 = Female	153	72.17
	1 = Male	59	27.83
Race	Asian-Latino/a	3	1.42
	Black-Latino/a	1	0.47
	Indigenous Latino/a	10	4.72
	Other	8	3.77
	White-Latino/a	190	89.62
Marital Status	1 = Married	86	40.57
	2 = Divorced	29	13.68
	3 = Widowed	11	5.19
	4 = Separated	2	0.94
	5 = Never married	74	34.91
	6 = Living with partner	9	4.25
	Missing	1	0.47
PANES	1 = Detached single-family housing	171	80.66
	2 = Townhouses, row houses, apartments, or condos of 2–3 stories	16	7.55
	3 = Mix of single-family residences and townhouses, row houses, apartments or condos	14	6.60
	4 = Apartments or condos of 4–12 stories	7	3.30
	5 = Apartments or condos of more than 12 stories	2	0.94
	Missing	2	0.94
Income	1 = Less than USD 10,000	13	6.13
	2 = USD 10,000–USD 24,999	15	7.08
	3 = USD 25,000–USD 34,999	23	10.85
	4 = USD 35,000–USD 49,999	41	19.34
	5 = USD 50,000–USD 74,999	48	22.64
	6 = USD 75,000–USD 99,999	21	9.91
	7 = USD 100,000–USD 149,999	23	10.85
	8 = USD 150,000–USD 199,999	12	5.66
	9 = USD 200,000 or more	6	2.83
	Missing	10	4.72
Education	1 = Never attended school or only attended kindergarten	0	0
	2 = Grades 1 through 4 (primary)	2	0.94
	3 = Grades 5 through 8 (middle school)	0	0
	4 = Grades 9 through 11 (some high school)	0	0
	5 = Grade 12 or GED (high school graduate)	17	8.02
	6 = 1 to 3 years after high school (some college, associate’s degree, or technical school)	59	27.83
	7 = College 4 years or more (college graduate)	61	28.77
	8 = Advanced degree (master’s, doctorate, etc.)	73	34.43
Mother’s Education	1 = Never attended school or only attended kindergarten	2	0.94
	2 = Grades 1 through 4 (primary)	29	13.68
	3 = Grades 5 through 8 (middle school)	20	9.43
	4 = Grades 9 through 11 (some high school)	19	8.96
	5 = Grade 12 or GED (high school graduate)	48	22.64
	6 = 1 to 3 years after high school (some college, associate’s degree, or technical school)	43	20.28
	7 = College 4 years or more (college graduate)	33	15.57
	8 = Advanced degree (master’s, doctorate, etc.)	13	6.13
	Missing	5	2.36
Father’s Education	1 = Never attended school or only attended kindergarten	0	0
	2 = Grades 1 through 4 (primary)	21	9.91
	3 = Grades 5 through 8 (middle school)	28	13.21
	4 = Grades 9 through 11 (some high school)	17	8.02
	5 = Grade 12 or GED (high school graduate)	50	23.58
	6 = 1 to 3 years after high school (some college, associate’s degree, or technical school)	33	15.57
	7 = College 4 years or more (college graduate)	39	18.40
	8 = Advanced degree (master’s, doctorate, etc.)	18	8.49
	Missing	6	2.83
HbA1c			
	1 = Diabetes	10	4.72
	2 = Normal	144	67.92
	3 = Prediabetes	55	25.94
	missing	3	1.42

**Table 3:** Correlations between metabolic health indicators HbA1c, BMI, WHR) and continuous socio-behavioral variables (*N* = 212).

	HbA1c	BMI	WHR	Age	Overall Health	Quality Of Life	Physical Health	Physical Performance	Instrumental Social Support Score	MSPSS Total Score	Neighborhood Physical Disorder Score	Cortisol
HbA1c	1.00	--	--	--	--	--	--	--	--	--	--	--
BMI	0.30 ***	1.00	--	--	--	--	--	--	--	--	--	--
WHR	0.29 ***	0.17 *	1.00	--	--	--	--	--	--	--	--	--
Age	0.31 ***	0.36 ***	0.31 ***	1.00	--	--	--	--	--	--	--	--
Overall Health	−0.22 **	−0.42 ***	−0.11	−0.13	1.00	--	--	--	--	--	--	--
Quality Of Life	−0.14 *	−0.36 ***	−0.11	−0.09	0.70 ***	1.00	--	--	--	--	--	--
Physical Health	−0.16 *	−0.43 ***	−0.11	−0.01	0.80 ***	0.74 ***	1.00	--	--	--	--	--
Physical Performance	−0.24 ***	−0.23 ***	−0.09	−0.10	0.26 ***	0.33 ***	0.22 **	1.00	--	--	--	--
Instrumental Social Support Score	−0.07	−0.14 *	−0.19 **	−0.15 *	0.29 ***	0.35 ***	0.27 ***	0.14 *	1.00	--	--	--
MSPSS Total Score	−0.15 *	−0.09	−0.22 **	0.00	0.27 ***	0.34 ***	0.29 ***	0.05	0.51 ***	1.00	--	--
Neighborhood Physical Disorder Score	0.06	0.16 *	0.20 **	0.04	−0.11	−0.20 **	−0.14 *	−0.11	−0.26 ***	−0.30 ***	1.00	--
Cortisol	0.06	0.1	0.03	−0.03	0.01	0.06	0.01	0.04	0.02	0.09	−0.12	1.00
Missing	3	0	0	0	0	0	0	0	0	0	1	3
Mean (sd)	5.65 (0.93)	29.99 (7.36)	0.88 (0.10)	43.45 (15.36)	3.38 (0.92)	3.60 (0.86)	3.21 (0.98)	4.67 (0.73)	4.00 (0.99)	5.80 (1.26)	1.55 (0.48)	9.55 (37.17)

Note: Pearson correlation analysis for continuous variables was performed. *p* < 0.001 ***; *p* < 0.01 **; *p* < 0.05 *.

**Table 4:** Correlation between health outcome variables (HbA1c, BMI, WHR) and ordinal socio-behavioral variables (*N* = 212).

	HbA1c	BMI	WH Ratio	Income	Education	Mother’s Education	Father’s Education
HbA1c	1.00	--	--	--	--	--	--
BMI	0.30 ***	1.00	--	--	--	--	--
WHR	0.29 ***	0.17 *	1.00	--	--	--	--
Income	0.02	−0.12	0.13	1.00	--	--	--
Education	−0.02	−0.13	0.12	0.37 ***	1.00	--	--
Mother’s Education	−0.32 ***	−0.23 ***	−0.27 ***	−0.01	0.02	1.00	--
Father’s Education	−0.34 ***	−0.30 ***	−0.28 ***	0.03	0.06	0.61 ***	1.00
*n*	209	212	212	202	212	207	206
Missing	3	0	0	10	0	5	6

Note: Pearson correlation analysis was performed to examine association between HbA1c, BMI, and WHR. Spearman correlation analysis was performed for all other correlations reported. *p* < 0.001 ***; *p* < 0.05 *.

**Table 5:** Correlation analysis among 15 biomarkers (*N* = 212).

	IL.1a	IL.1b	IL.1ra	IL.2	IL.4	IL.5	IL.6	IL.7	IL.8	IL.10	IL.12p40	IL.12p70	IL.13	IL.15	IL.17f
IL.1a	1.00	--	--	--	--	--	--	--	--	--	--	--	--	--	--
IL.1b	0.59 ***	1.00	--	--	--	--	--	--	--	--	--	--	--	--	--
IL.1ra	0.50 ***	0.60 ***	1.00	--	--	--	--	--	--	--	--	--	--	--	--
IL.2	0.46 ***	0.87 ***	0.55 ***	1.00	--	--	--	--	--	--	--	--	--	--	--
IL.4	0.40 ***	0.54 ***	0.44 ***	0.54 ***	1.00	--	--	--	--	--	--	--	--	--	--
IL.5	0.72 ***	0.60 ***	0.48 ***	0.48 ***	0.32 ***	1.00	--	--	--	--	--	--	--	--	--
IL.6	0.37 ***	0.64 ***	0.49 ***	0.59 ***	0.40 ***	0.39 ***	1.00	--	--	--	--	--	--	--	--
IL.7	0.18 *	0.24 **	0.18 *	0.41 ***	0.13	0.13	0.13	1.00	--	--	--	--	--	--	--
IL.8	0.55 ***	0.46 ***	0.53 ***	0.26 **	0.27 ***	0.38 ***	0.46 ***	0.17 *	1.00	--	--	--	--	--	--
IL.10	0.31 ***	0.47 ***	0.33 ***	0.43 ***	0.24 ***	0.44 ***	0.44 ***	0.24 ***	0.35 ***	1.00	--	--	--	--	--
IL.12p40	0.36 ***	0.53 ***	0.33 ***	0.49 ***	0.34 ***	0.41 ***	0.59 ***	0.31 ***	0.48 ***	0.49 ***	1.00	--	--	--	--
IL.12p70	0.45 ***	0.81 ***	0.42 ***	0.81 ***	0.56 ***	0.48 ***	0.68 ***	0.13	0.32 ***	0.38 ***	0.55 ***	1.00	--	--	--
IL.13	0.47 ***	0.76 ***	0.44 ***	0.78 ***	0.69 ***	0.35 ***	0.56 ***	0.13	0.35 ***	0.32 ***	0.46 ***	0.81 ***	1.00	--	--
IL.15	0.43 ***	0.67 ***	0.51 ***	0.52 ***	0.37 ***	0.46 ***	0.66 ***	0.20 **	0.55 ***	0.56 ***	0.53 ***	0.54 ***	0.51 ***	1.00	--
IL.17f	0.68 ***	0.61 ***	0.49 ***	0.47 ***	0.36 ***	0.62 ***	0.44 ***	0.30 ***	0.50 ***	0.56 ***	0.46 ***	0.41 ***	0.44 ***	0.57 ***	1.00

Note: Pearson correlation analysis for 15 biomarkers was performed. *p* < 0.001 ***; *p* < 0.01 **; *p* < 0.05 *.

## Data Availability

The original data presented in the study are openly available in Mendeley Data at https://data.mendeley.com/datasets/9rpk5p2nnc/1 (accessed on 28 February 2025).

## Supplementary Materials

The following supporting information can be downloaded at: https://www.mdpi.com/article/10.3390/ijerph22040647/s1. Figure S1. Heatmap for Correlations Between Metabolic Health Indicators (HbA1c, BMI, WHR) and Continuous Socio-Behavioral Variables (N = 212); Figure S2. Heatmap of Correlation Between Health Outcome Variables (HbA1c, BMI, WHR) and Ordinal Socio-Behavioral Variables (N = 212); Figure S3. Heatmap of Pearson Correlation Analysis Among 15 Biomarkers (N = 212).

## Abbreviations

The following abbreviations are used in this manuscript:

HbA1c	Hemoglobin a1c
BMI	Body Mass Index
WHR	Waist Hip Ratio
T1D	Type 1 Diabetes
T2D	Type 2 Diabetes
