# Supplementary material for: Exploring Socio-Behavioral Correlates of Metabolic and Inflammatory Risk in a University Sample Residing Along the U.S./Mexico Border: A Pilot Study Concomitantly Collecting Survey Data, Blood and Hair Samples, and Physical Measures
Source: Int J Environ Res Public Health. 2025 Apr 20;22(4):647. doi: 10.3390/ijerph22040647 (PMC12026700; doi:10.3390/ijerph22040647)
Supplement: Supplementary file 1 [file ijerph-22-00647-s001.zip › ijerph-3529535-supplementary.pdf]

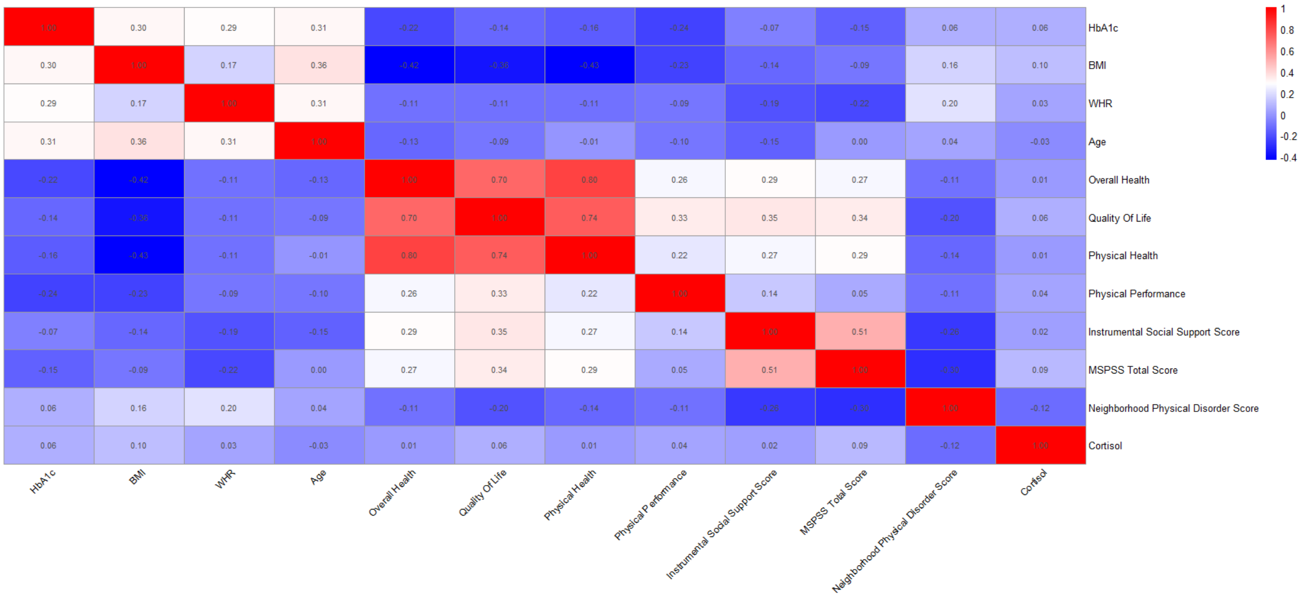

Figure S1: Heatmap for Correlations Between Metabolic Health Indicators (HbA1c, BMI, WHR) and Continuous Socio-Behavioral Variables (*N* = 212)

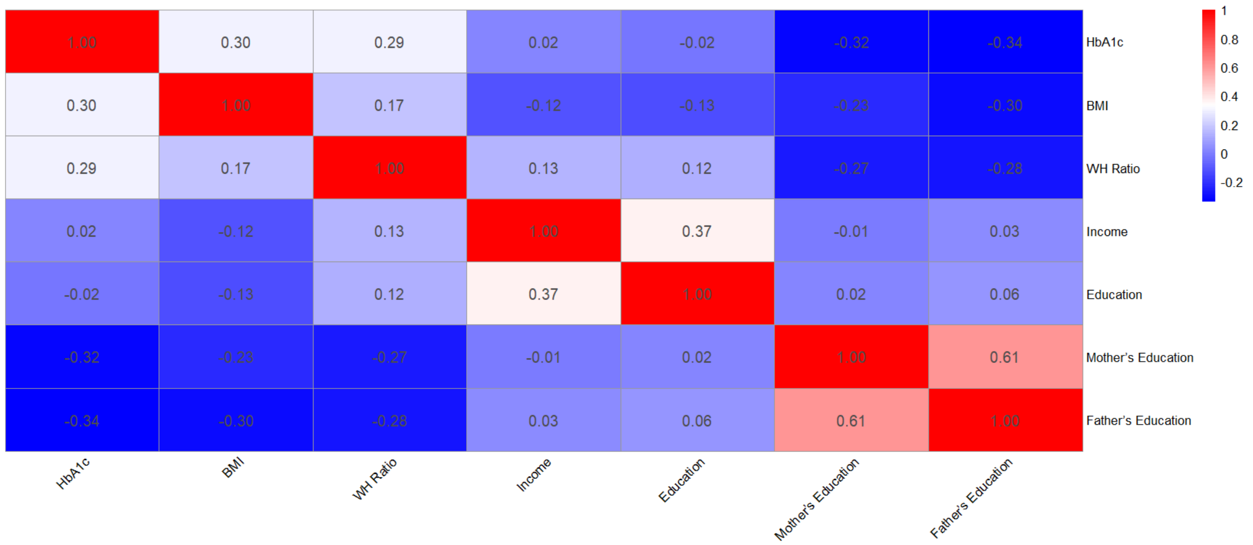

Figure S2: Heatmap of Correlation Between Health Outcome Variables (HbA1c, BMI, WHR) and Ordinal Socio-Behavioral Variables (*N* = 212)

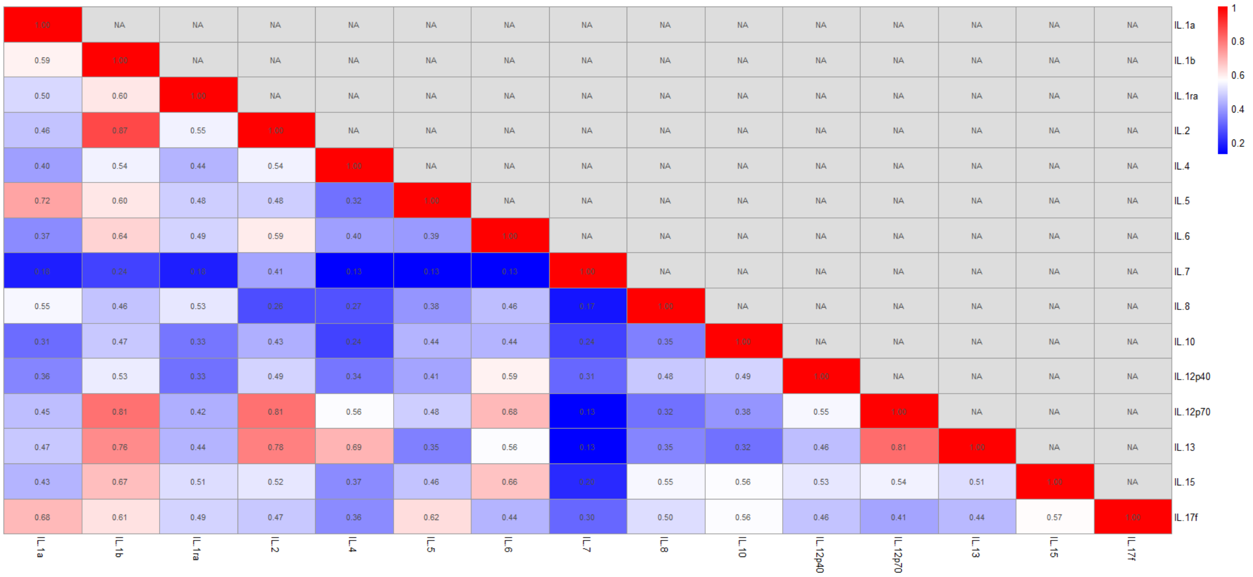

Figure S3: Heatmap of Pearson Correlation Analysis Among 15 Biomarkers (*N* = 212)
